# Different Effects of His‐Au NCs and MES‐Au NCs on the Propagation of Pseudorabies Virus

**DOI:** 10.1002/gch2.201800030

**Published:** 2018-06-25

**Authors:** Chenchen Feng, Puxian Fang, Yanrong Zhou, Lingzhi Liu, Liurong Fang, Shaobo Xiao, Jiangong Liang

**Affiliations:** ^1^ College of Science State Key Laboratory of Agricultural Microbiology Huazhong Agricultural University Wuhan 430070 P. R. China; ^2^ College of Veterinary Medicine State Key Laboratory of Agricultural Microbiology Huazhong Agricultural University Wuhan 430070 P. R. China

**Keywords:** antiviral activity, gold nanoclusters, proliferation, pseudorabies virus, surface modification

## Abstract

In a previous work, gold nanoclusters (Au NCs) are found to inactivate RNA virus, but the effect of surface modification of Au NCs on its proliferation is still largely unknown. Here, the effect of surface modification of Au NCs on the proliferation of pseudorabies virus (PRV) by synthesizing two types of gold clusters with different surface modification, histidine stabilized Au NCs (His‐Au NCs) and mercaptoethane sulfonate and histidine stabilized Au NCs (MES‐Au NCs), is investigated. His‐Au NCs rather than MES‐Au NCs could strongly inhibit the proliferation of PRV, as indicated by the results of plaque assay, confocal microscopic analysis, Western blot assay, and quantitative real‐time polymerase chain reaction (PCR) assay. Further study reveals that His‐Au NCs perform the function via blockage of the viral replication process rather than the processes of attachment, penetration, or release. Additionally, His‐Au NCs are found to be mainly localized to nucleus, while MES‐Au NCs are strictly distributed in cytoplasm, which may explain why His‐Au NCs can suppress the proliferation of PRV, but not MES‐Au NCs. These results demonstrate that surface modification plays a key role in the antiviral effects of Au NCs and a potential antiviral agent can be developed by changing the Au NC surface modification.

## 1 Introduction

With the development of nanotechnology, there are increasing numbers of reports on the antiviral activity of nanoparticles.^1^ Functional nanoparticles have been reported to affect the replication of virus, such as porous silicon nanoparticles, graphene oxide, carbon dots, quantum dots, and selenium nanoparticles.^2^ Furthermore, metal nanoparticles, such as silver nanoparticles, gold nanoparticles, magnesium oxide nanoparticles, titanium dioxide nanoparticles, and cuprous oxide nanoparticles, have also been confirmed to possess antiviral property to some extent.^3^ Meanwhile, functionalized nanoparticles were reported to have stronger antiviral activity. For instance, the silver nanoparticle modified with oseltamivir significantly inhibited H1N1 influenza virus infection via reducing the accumulation of reactive oxygen species (ROS).^4^ Multivalent sialic‐acid‐functionalized gold nanoparticles showed high inhibition activity against influenza virus infection.[[qv: 3d]] In our previous work, the CdTe quantum dots and carbon dots were demonstrated to possess antiviral activity against pseudorabies virus (PRV) to a certain extent.[[qv: 2e,5]]

Different from the gold nanoparticles (Au NPs), gold nanoclusters (Au NCs) are a new class of fluorophores that usually consist of various amounts of atom, ranging from a few to hundred, and Au NCs show two typical features.^6^ First, the size of Au NCs (usually less than 3 nm) approaches to the Fermi wavelength of electrons, which is much smaller than that of Au NPs. Second, Au NCs rather than Au NPs exhibit fluorescence in the visible to near‐infrared (NIR) region. Water‐soluble Au NCs have attracted increasing attention due to their excellent fluorescent properties, good biocompatibility, and well‐functionalized structure.^7^ Moreover, the synthetic methods of Au NCs are characterized by simplicity and diversity. For instance, Au NCs can be synthesized using various ligands including protein, peptide, DNA, and amino acid.^8^ Therefore, Au NCs have been widely applied to biosensing, bioimaging, drug delivery, tumor therapy, and so on.^9^ In a recent report, gold nanoclusters were used as nanocarriers to deliver the cas9 protein and the corresponding sgRNA plasmid into the cell nuclei, leading to reduction in the expression of the target gene *plk1* in the tumor and the inhibition of melanoma growth.^10^ Besides, Au NCs cross‐linked by cationic polymers could be self‐assembled into larger nanoparticles to improve the cellular uptake and drug delivery efficiency, and showed potential applications in theranostics.^11^ Previous studies have demonstrated the huge potential of Au NCs in nanomedicine, but the effect of Au NCs on virus remains to be explored.

A previous study has proved that the mercaptoethane sulfonate (MES) could improve the antiviral effect of nanoparticles after functionalizing the surface of nanoparticles, for MES is an analogue of heparin sulfate (HS), a cellular receptor related to virus infection.^12^ In this research, we synthesized MES and histidine‐stabilized Au NCs (MES‐Au NCs) by ligand exchange between MES and His from His‐stabilized Au NCs (His‐Au NCs), and compared the influence of MES‐Au NCs and His‐Au NCs on the propagation of PRV, a virus that can cause the so‐called Aujeszky's disease and a massive economic loss in the swine industry due to its high mortality.^13^ Interestingly, we found that His‐Au NCs rather than MES‐Au NCs have a significant inhibitory effect on the replication of PRV by suppressing the replication process. These results may facilitate further research on Au NCs as an effective and safe specific inhibitor of virus.

## 2 Results and Discussion

### 2.1 Characterization of His‐Au NCs and MES‐Au NCs

**Figure 1:**
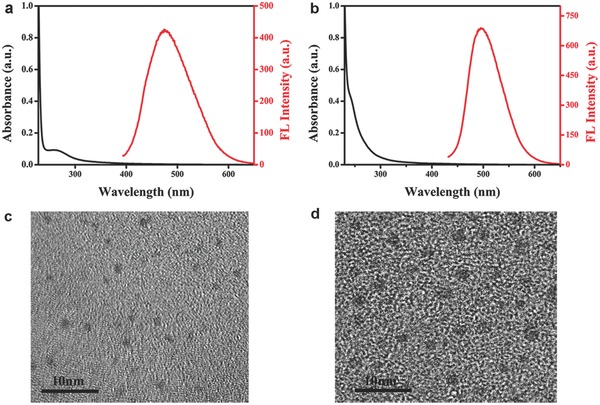
UV‐vis absorption spectrum and fluorescence emission spectrum of a) His‐Au NCs and b) MES‐ Au NCs. The HRTEM images of c) His‐Au NCs and d) MES‐ Au NCs.

The basic optical and morphological properties of His‐Au NCs and MES‐Au NCs were characterized and shown in **Figure** **1**. In Figure 1a, the UV‐vis spectra of His‐Au NCs showed a maximum absorption wavelength at 258 nm and a band edge at 455 nm. The obvious increase in the absorption spectra of His‐Au NCs below 310 nm indicated that the Au NCs did not have the surface plasmon resonance (SPR) peak as reported previously.^14^ Moreover, the His‐Au NCs exhibited a fluorescence emission peak at 475 nm with the excitation at 380 nm. However, the UV–vis absorption augmentation of MES‐Au NCs (Figure 1b) was sharply below 300 nm and the absorption above 585 nm was not observed, which may result from the molecular‐like properties of the MES‐Au NCs as previously reported.^15^ With the excitation wavelength at 410 nm, the MES‐Au NCs had a fluorescence emission peak at 498 nm. There was an obvious difference between the His‐Au NCs and MES‐Au NCs in the UV–vis absorption and fluorescence spectra. The morphology and size of His‐Au NCs and MES‐Au NCs were acquired from the high‐resolution transmission electron microscopy (HRTEM) image. As shown in Figure 1c,d, the average size of His‐Au NCs was 1.2 ± 0.2 nm with a regular distribution and a consistent shape, while that of MES‐Au NCs was 2.1 ± 0.4 nm, significantly larger than His‐Au NCs.

His‐Au NCs, MES‐Au NCs, and MES were further characterized by IR spectroscopy (Figure S1, Supporting Information). For His‐Au NCs, the peak at 3415 cm^−1^ was ascribed to the stretching vibration of O—H bond while the peaks at 3009 and 2887 cm^−1^ were attributed to the stretching vibration of N—H.^16^ Peaks at 2710 and 1635 cm^−1^ were assigned to the stretching vibration of C—H and C=O, respectively.^17^ The peaks at 1463 and 1148 cm^−1^ were due to the vibration of imidazole ring and the bending vibration of N—H on imidazole ring.^18^ The aforementioned peaks demonstrated the existence of amidogen, carboxyl, and imidazole ring on the surface of His‐Au NCs. For MES‐Au NCs, the peak at 3448 cm^−1^ was due to the stretching vibration of O—H and the peaks at 3008 and 2887 cm^−1^ were attributed to the stretching vibrations of N—H. Besides, the peaks at 2706, 1635, 1464, and 1147 cm^−1^ were assigned to the stretching vibrations of C—H, C=O, imidazole ring, and the bending vibration of N—H, respectively, confirming the existence of histidine on the surface of MES‐Au NCs. Furthermore, peaks at 1211 and 1180 cm^−1^ could be ascribed to the stretching vibration of S=O, demonstrating the existence of terminal sulfonic group and confirming the formation of MES‐conjugated MES‐Au NCs.^19^ Obviously, the S—H stretching vibration of MES at 2571 cm^−1^ disappeared after the formation of MES‐Au NCs, suggesting the combination of MES with Au NCs via the formation of Au—S bond.^20^ These results indicate that MES‐Au NCs were stabilized by histidine and MES together.

The time‐resolved fluorescence decay curves of His‐Au NCs and MES‐Au NCs are shown in Figure S2 (Supporting Information). The two unique decay times (1.74 and 6.54 ns) of His‐Au NCs could be well fitted with a double exponential function, while the decay time (8.27 ns) of MES‐Au NCs could fit a single exponential function. The longer decay time of MES‐Au NCs may be due to the electron donor of MES. These results further demonstrated the formation of MES‐Au NCs.^21^

The composition of His‐Au NCs and MES‐Au NCs was further investigated by X‐ray photoelectron spectroscopy (XPS) (Figure S3, Supporting Information). In the survey spectra of His‐Au NCs (Figure S3a, Supporting Information) and MES‐Au NCs (Figure S3b, Supporting Information), the peaks at 530.6, 400.13, 284.78, and 84.77 eV/531.15, 400.1, 284.77, 167.77, and 84.04 eV were attributed to O1s, N1s, C1s, and Au4f or O1s, N1s, C1s, S2p, and Au4f, respectively.^22^ The existence of S and N element further indicated that the MES‐Au NCs were stabilized by histidine and MES. In the Au4f spectrum of His‐Au NCs and MES‐Au NCs (Figure S3c, Supporting Information), after partial replacement of histidine with MES, the peak of Au4f_7/2_ shifted from 84.7 to 84.1 eV and the peak of Au4f_5/2_ moved from 88.5 to 87.7 eV causing the transformation of the binding energy of Au4f.^23^ In Figure S3d (Supporting Information), no S2p was detected in His‐Au NCs, and the peaks in the S2p spectrum of MES‐Au NCs at 163.2 and 167.7 eV should be ascribed to the sulfur from the sulfhydryl group and sulfonate group, demonstrating the successful formation of MES‐Au NCs.^19^

### 2.2 Cytotoxicity Assay

**Figure 2:**
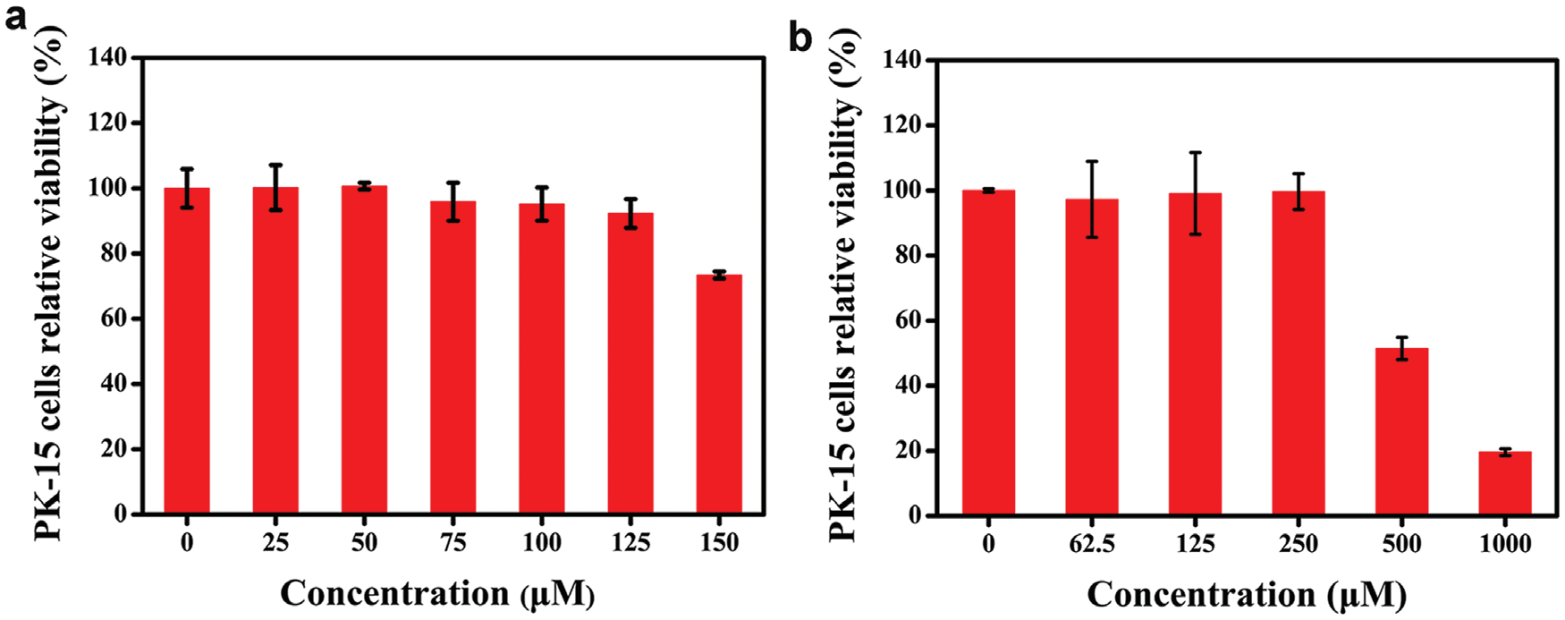
The cytotoxicity of different concentrations of His‐Au NCs and MES‐Au NCs by MTT assay. PK‐15 cells were incubated with a) His‐Au NCs and b) MES‐Au NCs for 24 h. Error bars represent the standard deviation from three independent experiments.

The cytotoxicity of His‐Au NCs is different from that of MES‐Au NCs. Zhang et al. have reported that the cytotoxicity of His‐Au NCs is higher than that of glutathione and histidine‐capped Au NCs (GSH‐Au NCs).^24^ In this experiment, a different range of concentrations (50 and 250 × 10^−6^ m for His‐Au NCs and MES‐Au NCs) was used as reported in previous literature.^24^ The toxicity of His‐Au NCs and MES‐Au NCs on Porcine kidney (PK‐15) cells was evaluated by 3‐(4,5‐dimethyl‐2‐yl)‐2,5‐diphenyltetrazolium bromide (MTT) assay as described previously.^5^ In **Figure** **2**a, His‐Au NCs showed little or no effect on the activity of PK‐15 cells in the concentration range of 25–150 × 10^−6^ m and the viability of the cells was more than 95% at 50 × 10^−6^ m, which was therefore utilized to explore the effect of His‐Au NCs on virus proliferation in the subsequent experiments. Additionally, the mortality rate of the PK‐15 cells was less than 3% at the 250 × 10^−6^ m concentration of MES‐Au NCs (Figure 2b), thus 250 × 10^−6^ m concentration of MES‐Au NCs was used for further experiments.

### 2.3 Effects of His‐Au NCs and MES‐Au NCs on PRV Proliferation

Recently, we have found that GSH‐Au NCs showed no effect on PRV.^25^ However, previous study has demonstrated that gold and silver nanoparticles capped with MES that mimicked the cell surface receptor HS could inhibit HSV‐1 infection.^26^ Mechanically, the HS mimics may compete for binding viruses to target cells, leading to blockage of the interaction of virus with cell surface receptor.[[qv: 26b]] Therefore, in this experiment, MES‐Au NCs were first synthesized through partial exchange of the histidine on the surface of His‐Au NCs via formation of Au—S bond. Then, the antiviral activity of His‐Au NCs and MES‐Au NCs on PRV was investigated by confocal microscopic analysis using the recombinant PRV expressing fluorescent protein (GFP‐PRV).^27^

**Figure 3:**
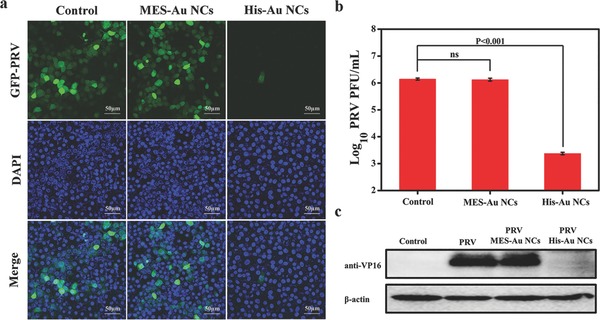
The antiviral activity of His‐Au NCs versus MES‐Au NCs. a) The fluorescent images assay of PK‐15 cells infected with GFP‐expressing PRV in the presence or absence of His‐Au NCs and MES‐Au NCs for 12 h, with nuclei counterstained with DAPI (blue); the scale bars are 50 µm. b) The titer of PRV was determined in the presence or absence of His‐Au NCs and MES‐Au NCs for 12 h by plaque assay. c) The expression level of PRV VP16 protein was detected in the presence or absence of His‐Au NCs MES‐Au NCs by Western blot assay. β‐actin was used as a loading control. Error bars represent the standard deviation from three independent experiments.

In **Figure** **3**a, obvious green fluorescence signals could be observed in GFP‐PRV infected cells at 12 h postinfection. However, specific green fluorescence signals significantly decreased in His‐Au NCs treated cell group compared to the control group. Interestingly, the treatment of MES‐Au NCs showed no significant negative effect on the proliferation of GFP‐PRV compared to the control group under the same experimental conditions. According to a previous report, PRV can also bind to other surface receptors other than HS.^28^ Moreover, it has been reported that HSV‐1 and PRV can initiate the infection by using a common and limited cell surface component rather than HS in Vero cells.^28^ These results may explain why MES‐Au NCs do not affect the proliferation of GFP‐PRV.

To further verify that His‐Au NCs rather than MES‐Au NCs have the antiviral activity against the wild‐type PRV, plaque assays were performed to detect the virus titers with or without the treatment of 50 × 10^−6^ m His‐Au NCs and 250 × 10^−6^ m MES‐Au NCs. As indicated in Figure 3b, the virus titers from the control group reached up to ≈10^6^ plaque forming units (PFU) mL^−1^ at 12 h postinfection (hpi). However, the virus titers obviously decreased under the treatment of His‐Au NCs rather than MES‐Au NCs compared to the control group.

PRV VP16 is a tegument protein encoded by a UL48 gene, which is highly conserved in *Alphaherpesvirinae*. VP16 has been extensively studied and found to play multiple functions in enhancing the expression levels of viral immediate‐early genes and viral egress.^29^ To further confirm the effect of His‐Au NCs on PRV infection, the Western blot assay was performed to detect the expression of VP16 protein in virus infected cells with or without the treatment of His‐Au NCs and MES‐Au NCs at 12 hpi. The results demonstrated that the expression of VP16 was obviously observed in the control group (Figure 3c). However, a remarkably decreased expression level of VP16 was detected under the treatment of His‐Au NCs, and no significant difference was observed between the treatment of MES‐Au NCs and the control group. These results were completely consistent with the data from confocal microscopic analysis (Figure 3a) and plaque assays (Figure 3b). Taken together, these results revealed that His‐Au NCs have a strong ability to inhibit the PRV proliferation, but not MES‐Au NCs.

### 2.4 Influence of His‐Au NCs on PRV Replication Cycle

**Figure 4:**
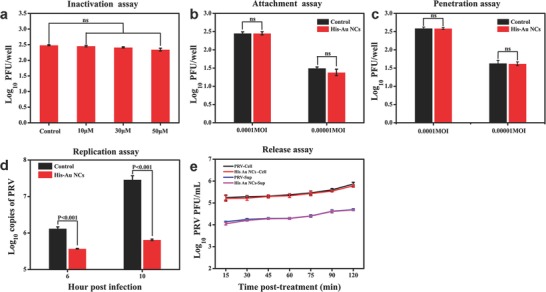
The effect of 50 × 10^−6^ m His‐Au NCs on PRV infection under different treatment conditions. PK‐15 cells infected with PRV (MOI = 1) were treated with His‐Au NCs under different treatment conditions. a) Virus inactivation, b) attachment assay, c) penetration assay, d) replication assay, and e) release assay. e) The virus titer from the cells and cellular supernatant at indicated time points. Correlation analyses were performed by Graphpad Prism 5.0.

It is well known that viral replication cycle includes multiple stages such as viral attachment, penetration, replication, and release of progeny virion.^30^ To further investigate which stage was affected by His‐Au NCs during the PRV life cycle, the action mode of His‐Au NCs was investigated by inactivation assay, attachment assay, penetration assay, replication assay, and release assay. In the inactivation assay (**Figure** **4**a), the virus titers under the treatment of different concentrations of His‐Au NCs (0, 10, 30, and 50 × 10^−6^ m) showed no significant difference from each other. In the attachment assay (Figure 4b) and penetration assay (Figure 4c), plaque analysis also showed no significant difference in the virus titers between the treatment group of different concentrations of His‐Au NCs and the control group, suggesting His‐Au NCs have no influence on attachment and penetration. A previous study revealed that, after PRV infection, the viral genome is released and enters the nucleus via the nuclear pore, followed by the viral genome DNA replication.^31^ Therefore, as described previously, the influence of His‐Au NCs on the replication process was confirmed by detecting the viral DNA synthesis at different time points via quantitative real‐time PCR assay. As shown in Figure 4d, the copies of PRV significantly increased in untreated cells from 6 to 10 hpi, while His‐Au NCs obviously depressed this proliferation tendency. At 10 hpi, the level of inhibition was up to ≈2 orders of magnitude. These results suggested that His‐Au NCs could interfere with the viral genome DNA replication. To further identify the influence of His‐Au NCs on the viral release from PK‐15 cells, plaque assays were carried out. As shown in Figure 4e, the virus titers from both the cell and supernatant showed no obvious difference between the control group and the His‐Au NCs treatment group even at 120 min post‐treatment, attesting that the His‐Au NCs did not affect PRV release. The aforementioned experimental results demonstrated that His‐Au NCs inhibit the proliferation of PRV via obstructing the viral replication rather than other processes during PRV proliferation.

### 2.5 The Mechanism Research on the Inhibition of PRV by His‐Au NCs

Previous studies have found that the surface charge and groups have influence on the internalization process of nanomaterials into cells and further affect their cellular location.^32^ Furthermore, the cellular location of Au NCs may be related to the different antiviral performance of various materials.[[qv: 2d]] To explore the inhibitory mechanism of His‐Au NCs on PRV, we investigated the cellular locations of His‐Au NCs and MES‐Au NCs.

**Figure 5:**
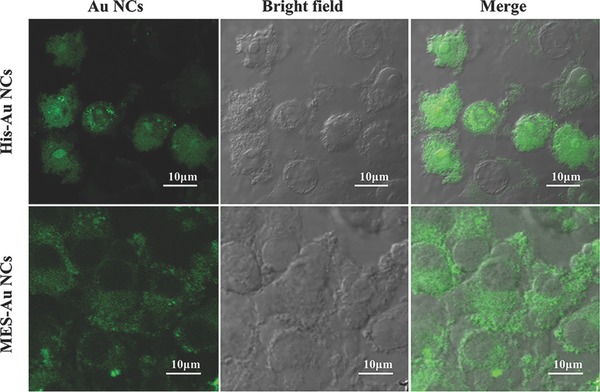
Confocal fluorescence images of PK‐15 cells incubated with His‐Au NCs or MES‐Au NCs for 12 h with an excitation wavelength at 405 nm. Scale bars: 10 µm.

To test the hypothesis that the different performance of His‐Au NCs and MES‐Au NCs is related to their distribution in cells, the bioimaging assay was conducted. In the experiments of the effects of Au NCs on PRV proliferation, the concentrations of His‐Au NCs and MES‐Au NCs were 50 and 250 × 10^−6^ m, respectively. The same concentrations of His‐Au NCs and MES‐Au NCs were also used for exploring the inhibitory mechanism on PRV. After incubation with His‐Au NCs (50 × 10^−6^ m) or MES‐Au NCs (250 × 10^−6^ m) for 12 h, PK‐15 cells were observed under the confocal laser scanning microscope at the excitation wavelength of 405 nm. As shown in **Figure** **5**, His‐Au NCs were mainly localized to nucleus, while MES‐Au NCs were strictly distributed in cytoplasm. Considering that the cellular uptake properties of the Au NCs are decided by their structure and character such as size, surface functional groups, surface charge, and so on, the size and surface functional groups were investigated in our experiment. In Figure S4 (Supporting Information), the zeta potentials of the His‐Au NCs and MES‐Au NCs were −12.3 ± 1.2 and −23.7 ± 1.4 mV, respectively. The zeta potential of the MES‐Au NCs was more negatively charged than that of His‐Au NCs, indicating it was more difficult for MES‐Au NCs to enter the cell because of the electrostatic interactions. Besides, the much larger size of MES‐Au NCs (2.1 ± 0.4 nm) than His‐Au NCs (1.2 ± 0.2 nm) could also affect the entry of MES‐Au NCs into nucleus. The results were in line with data from Figure 5. Therefore, His‐Au NCs could enter cell nucleus, while it was very difficult for MES‐Au NCs to reach the cell nucleus from cytoplasm. In our previous work, the effect of various surface charges of carbon dots (CDs) on PK‐15 cells was investigated, and cyan‐fluorescent CDs (c‐CDs) with a neutral surface charge were found to enter nucleus, but not blue‐fluorescent CDs (d‐CDs) with a negative surface charge, leading to the different antiviral performance of c‐CDs and d‐CDs.[[qv: 2d]] A previous study also indicated that the replication of DNA viruses (PRV) mainly takes place in nucleus.^33^ Therefore, the different location of His‐Au NCs and MES‐Au NCs in cells is likely to indicate that His‐Au NCs rather than MES‐Au NCs specifically inhibit the proliferation of PRV.

## 3 Conclusion

This is the first report on the antiviral activities of His‐Au NCs and MES‐Au NCs on PRV as a DNA virus model. In the study, we found that surface charge plays a crucial role in the antiviral effect of gold cluster. Interestingly, His‐Au NCs with little negative charge rather than MES‐Au NCs with much negative charge were verified to have obvious antiviral activity against PRV at a noncytotoxic concentration via a series of detection assays, such as fluorescent protein assay using GFP‐PRV, confocal microscopic analysis, plaque assay, quantitative real‐time PCR assay, and Western blot assay. Furthermore, the results of cellular imaging revealed that His‐Au NCs are almost localized in cell nucleus, where the genome replication of PRV takes place, while MES‐Au NCs are totally localized in the cytoplasm. These results suggest the potential similar location of His‐Au NCs with PRV during the replication process, but not MES‐Au NCs, which may account for the difference between His‐Au NCs and MES‐Au NCs in their antiviral effect on PRV. These encouraging findings could deepen our understanding of the relationship between Au NCs and viruses and promote further research on Au NCs as a novel antiviral agent.

## 4 Experimental Section

*Reagents*: l‐Histidine, MES, bovine serum albumin (BSA), and MTT (98%) were obtained from Aladdin Regent Co. Ltd. HAuCl_4_·4H_2_O was supplied from Shanghai Chemical Reagent Co. Ltd. Fetal bovine serum (FBS) and Dulbecco's modified Eagle's medium (DMEM) were purchased from Invitrogen Regent Co. Ltd. Agarose was acquired from Promega Co. Ltd. Some common reagents were purchased from Sinopharm Chemical Regent Co. Ltd. The deionized water with a resistivity of 18.2 MΩ cm was used in this study. All chemicals and solvents were of analytical grade or better and used without further purification.

*Apparatus*: The UV–vis absorption spectra from 650 to 230 nm were recorded on a UV‐2450 spectrophotometer (Shimadzu, Japan). Fluorescence measurements and fluorescence lifetime measurements were performed using an RF‐5301 PC spectrofluorometer (Shimadzu, Japan) and an FLS920 Spectro‐Fluorimeter (Edinburgh Instruments Ltd., England), respectively. Fourier transform infrared spectra (FTIR) were obtained to identify the molecular structures of Au NCs with a Nicolet Avatar‐330 spectrometer (Thermo Nicolet, USA) through the KBr pellet technique. Inductively coupled plasma atomic emission spectrometry (ICP‐AES) was performed on a Perkin Elmer Optima 5300DV (Perkin Elmer, USA). The size and morphology of Au NCs were recorded on a HRTEM (Tecnai G2 F30, FEI, USA). XPS was conducted with an Escalab 250Xi photoelectron spectrometer (Thermo, USA) equipped with monochromatic Al Kα radiation. The zeta potential was obtained through the Zetasizer Nano ZS90 dynamic light scattering (DLS) system (Malvern, England). The ZEISS LSM 800 with Airyscan (Carl Zeiss AG, Germany) was used to take the confocal fluorescence images.

*Synthesis of His‐Au NCs and MES‐Au NCs*: His‐Au NCs were synthesized as reported previously.^34^ Briefly, the newly prepared aqueous solution of histidine (7.5 mL, 0.1 m) was mixed with HAuCl_4_ solution (2.5 mL, 10 × 10^−3^ m) at room temperature for 2 h without stirring to obtain the His‐Au NCs, then the MES‐Au NCs were acquired after the addition of the freshly prepared MES (10 mL, 60 × 10^−3^ m) solution and incubation overnight. The as‐prepared His‐Au NCs and the MES‐Au NCs solutions were stored at 4 °C for further use.

*Cell Culture and Viruses*: PK‐15 cells were purchased from the American Type Culture Collection (ATCC No. CRL‐1223) and were grown in DMEM (Invitrogen) supplemented with 10% FBS 37 °C in a humidified 5% CO_2_ incubator. PRV isolate Ea (GenBank Accession No. KX423960) was propagated in PK‐15 cells.

*Cytotoxicity Assay*: In order to assess the cytotoxicity of His‐Au NCs and MES‐Au NCs on PK‐15 cells, MTT assay was performed as described previously.^5^ Initially, PK‐15 cells at 90% confluence were treated with the mixture solution (100 µL) of different concentrations of His‐Au NCs (0, 25, 50, 75, 100, 125, and 150 × 10^−6^ m) or MES‐Au NCs (0, 62.5, 125, 250, 500, and 1000 × 10^−6^ m) and DMEM containing 2% FBS at a 96‐well plate for 24 h at 37 °C in a humidified 5% CO_2_ atmosphere. Then MTT (5.0 mg mL^−1^) was added into the cells with 20 µL well^−1^, followed by incubation for 4 h at 37 °C. Subsequently, the cell supernatants were removed, followed by the addition of 150 µL DMSO to dissolve formazan crystals. Finally, the absorption values were measured at 570 nm with an enzyme linked immunosorbent assay (ELISA) microplate reader and then the cell viability percentage was calculated.

*The Effect of His‐Au NCs and MES‐Au NCs on PRV Propagation*: PK‐15 cells were cultured in 24‐well plates until 80–90% confluence, followed by incubation with 50 × 10^−6^ m His‐Au NCs or 250 × 10^−6^ m MES‐Au NCs for 2 h at 37 °C under 5% CO_2_. Afterward, the cells were infected with 1 multiplicity of infection (MOI) recombinant GFP‐PRV or wild‐type PRV which had been incubated with His‐Au NCs or MES‐Au NCs at 4 °C for 1 h. After infection, the supernatant was removed, and after washing twice with PBS, the cells were treated with DMEM containing 2% FBS with or without 50 × 10^−6^ m his‐Au NCs (250 × 10^−6^ m MES‐Au NCs). After incubation for 12 h in the presence or absence of 50 × 10^−6^ m His‐Au NCs or 250 × 10^−6^ m MES‐Au NCs, the as‐treated PK‐15 cells were subjected to image capture using a confocal laser scanning microscope, plaque assay, and Western blot assay.

*Plaque Assays*: The plaque assays were performed as described previously.^5^ Briefly, the monolayers of PK‐15 cells in six‐well plates were incubated with tenfold diluted virus samples (800 µL) with DMEM containing 2% FBS for 1 h at 37 °C, followed by removing the inoculum. After washing three times with DMEM, the cells were supplemented with 2× DMEM with 3% FBS mixed with 1.8% (w/v) low melting point agarose (Promega) at the rate of 1:1 and incubated for ≈2–3 d. Subsequently, the cells were stained with the neutral red dye mixed with PBS at the rate of 1:1 for 2 h at 37 °C. Virus titer was acquired by calculating the average plaque number from three independent experiments, and expressed as PFU mL^−1^.

*Western Blot Assay*: PK‐15 cells were treated as described above. Subsequently, at the indicated time, the cells were harvested using the method described previously.^35^ Briefly, the obtained cell lysates were mixed with 5× SDS protein loading buffer and boiled for 10 min at 95 °C, followed by separation by sodium dodecyl sulfate polyacrylamide gel electrophoresis (SDS‐PAGE) with 12% polyacrylamide gels. Then the separated protein was transferred to polyvinylidene fluoride (PVDF) membrane (Millipore), and the membrane was blocked with 5% skim milk in phosphate buffered saline with Tween‐20 (PBST). Primary antibodies against VP16 or β‐actin were incubated with the membrane for 2 h at room temperature, followed by incubation with horseradish peroxidase‐conjugated goat anti‐rabbit/mouse IgG (1:5000 dilution, in PBST) for 45 min at room temperature after washing three times with PBST. Finally, the membrane was visualized by enhanced chemiluminescence reagents (ELC; BIO‐RAD) after washing three times.

*Inactivation Assay*: (0.001 MOI) PRV was incubated with different concentrations of His‐Au NCs (0, 10, 30, and 50 × 10^−6^ m) for 1 h at 37 °C, followed by tenfold dilution. The monolayers of PK‐15 cells were first precooled at 4 °C for 30 min and then mixed with the above treated PRV at 4 °C for 2 h, followed by three washes with PBS. Finally, the plague assay was conducted to detect the virus titer using the method described above.

*Attachment Assay*: PK‐15 cells with 80–90% confluence were incubated at 4 °C for 30 min, followed by infection with (0.0001, 0.00001 MOI) PRV with or without 50 × 10^−6^ m His‐Au NCs at 4 °C for 2 h. Then the inoculum was removed and then the cells were washed with cooled DMEM. Finally, the plague assay was performed as described in the inactivation assay.

*Penetration Assay*: PK‐15 cells with 80–90% confluence were first incubated in 4 °C for 30 min and then infected with (0.0001, 0.00001 MOI) PRV in the presence or absence of 50 × 10^−6^ m His‐Au NCs. After three washes with PBS, the cells were incubated with DMEM containing 2% FBS with or without 50 × 10^−6^ m His‐Au NCs for another 30 min at 37 °C. Finally, the plague assay was performed as described in the inactivation assay.

*Replication Assay*: PK‐15 cells with the confluence of 80–90% were incubated with DMEM containing 2% FBS with or without 50 × 10^−6^ m His‐Au NCs at 37 °C for 2 h, followed by infection with PRV (1 MOI) at 37 °C for 1 h. After three washes with PBS, the cells were incubated with DMEM containing 2% FBS in the presence or absence of 50 × 10^−6^ m His‐Au NCs at 37 °C for 6 and 10 h. At the indicated time, the total DNA of cells was extracted using viral DNA kit (OMEGA) and subjected to quantitative real‐time PCR (qPCR) to amplify the PRV glycoprotein B gene (gB) by the SYBR green PCR assay (Applied Biosystems) with the Applied Biosystems ViiA 7 Real‐time PCR System (Life Technologies). The upstream and downstream primers gB‐F (5′‐GGTCACCCGCGTGCTGATC‐3′) and gB‐R (5′‐ACTACGAGGACTACAGCTACGTGC‐3′) were separately used to amplify a 65‐bp fragment of gB gene of PRV. The copies of PRV DNA were calculated based on the results for a standard tenfold plasmid dilution (from 10^1^ to 10^10^).

*Release Assay*: PK‐15 cells with 80–90% confluence were infected by (1 MOI) PRV for 1 h, followed by removal of the inoculum, addition of freshly prepared DMEM containing 2% FBS and incubation at 37 °C for 7 h. Then the cell supernatants were removed and the cells were maintained with DMEM containing 2% FBS with or without 50 × 10^−6^ m His‐Au NCs. Finally, the cell supernatant and cells were harvested separately at 15, 30, 45, 60, 75, 90, and 120 min post‐treatment and stored at −80 °C for further use. The plaque assay was performed to detect the virus titer as described above.

*Cellular Imaging*: The monolayer of PK‐15 cells was inoculated with DMEM containing 2% FBS with 50 × 10^−6^ m His‐Au NCs or 250 × 10^−6^ m MES‐Au NCs for 12 h at 37 °C. Then the cell image was obtained from the confocal laser scanning microscope at the excitation wavelength of 405 nm.

*Statistical Analysis*: The data were presented as mean ± standard deviation of three independent experiments. Statistical differences were determined by one‐way analysis of variance (ANOVA) using GraphPad Prism 5.0 software. Differences were considered to be statistically significant at *P* < 0.05.

## Conflict of Interest

The authors declare no conflict of interest.

## Supporting information

Supplementary
