# Supplementary material for: Different Effects of His‐Au NCs and MES‐Au NCs on the Propagation of Pseudorabies Virus
Source: Glob Chall. 2018 Jun 25;2(8):1800030. doi: 10.1002/gch2.201800030 (PMC6607262; doi:10.1002/gch2.201800030)
Supplement: Supplementary file 1 — Supplementary [file GCH2-2-1800030-s001.pdf]

Copyright WILEY-VCH Verlag GmbH & Co. KGaA, 69469 Weinheim, Germany, 2018.

Global Challenges
Open Access

Supporting Information

for *Global Challenges,* DOI: 10.1002/gch2.201800030

Different Effects of His-Au NCs and MES-Au NCs on the Propagation of Pseudorabies Virus

*Chenchen Feng, Puxian Fang, Yanrong Zhou, Lingzhi Liu, Liurong Fang, Shaobo Xiao, and Jiangong Liang**

DOI: 10.1002/gch2.201800030

Supporting Information

Different Effects of His-Au NCs and MES-Au NCs on the Propagation of Pseudorabies Virus

*Chenchen Feng‡, Puxian Fang‡, Yanrong Zhou, Lingzhi Liu, Liurong Fang, Shaobo Xiao, Jiangong Liang**

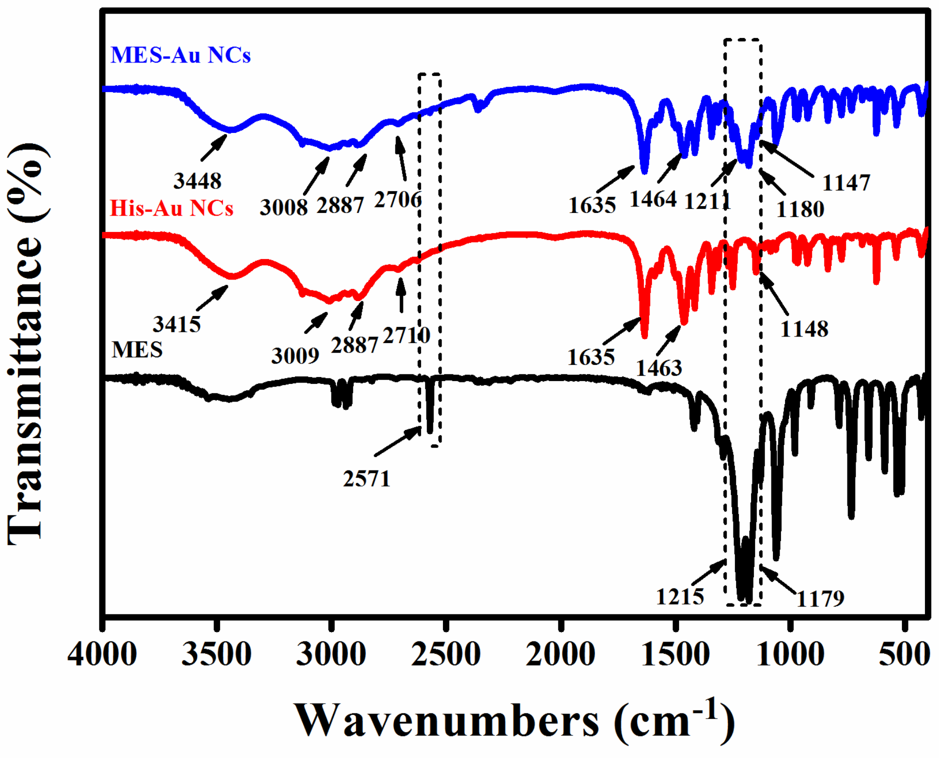

Figure S1. The FT-IR spectra of His-Au NCs, MES- Au NCs and MES.

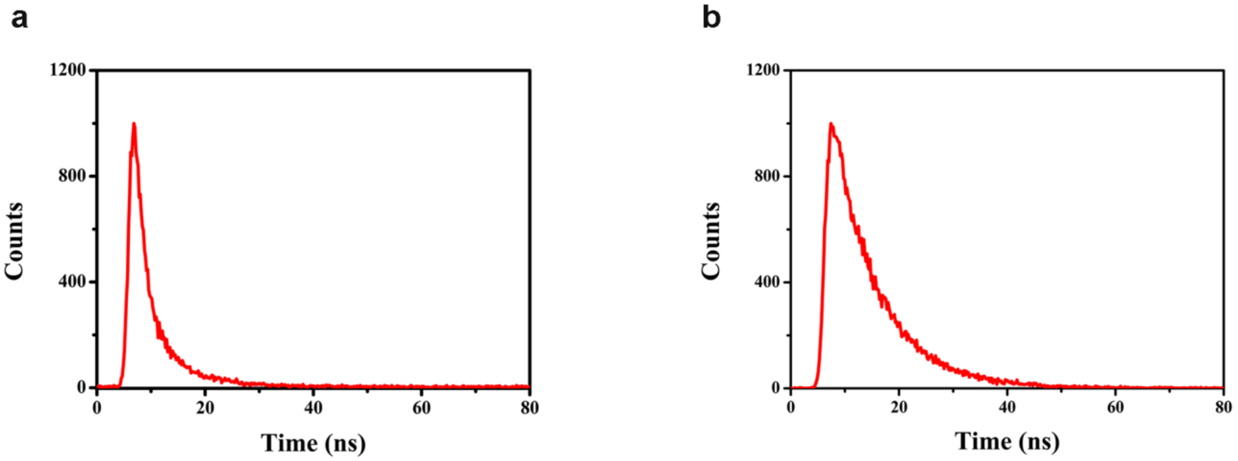

Figure S2. The fluorescence decay curves of His-Au NCs (a) and MES- Au NCs (b).

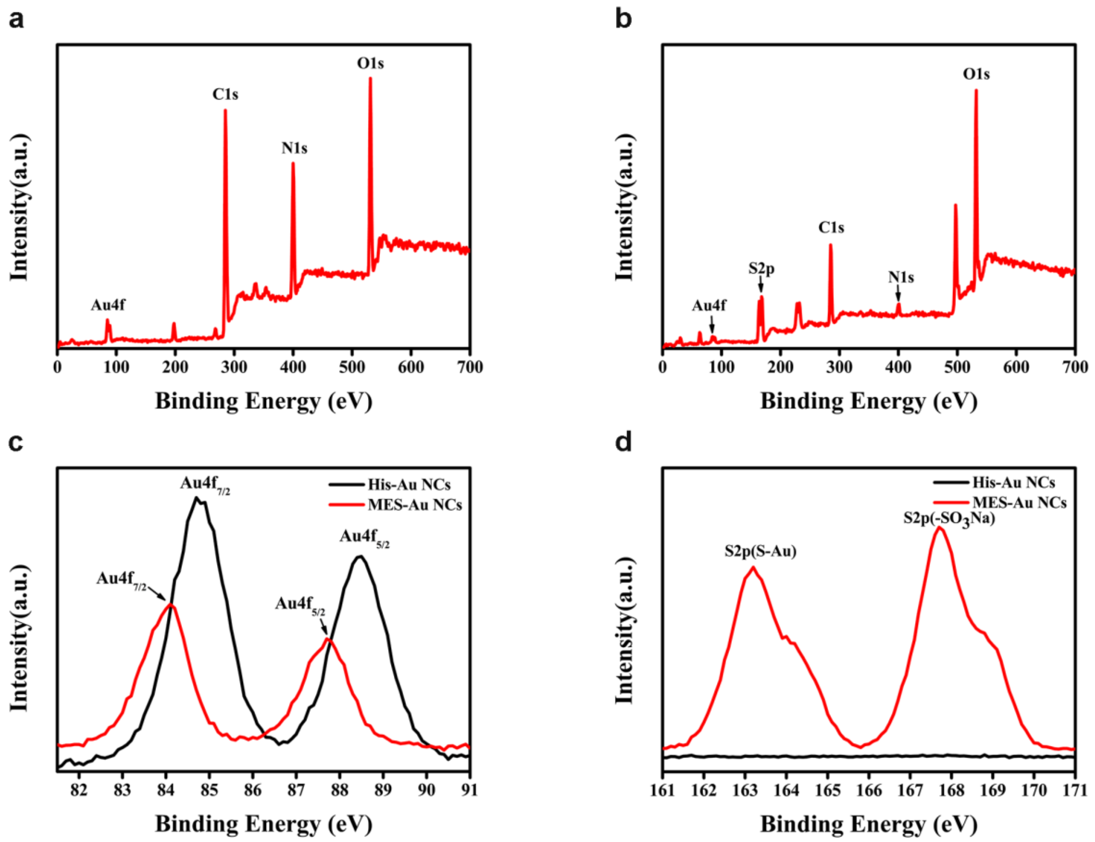

Figure S3. XPS spectra of (a) survey spectrum of His-Au NCs, (b) survey spectrum of MES-Au NCs, (c) Au4f region of His-Au NCs and MES-Au NCs, and (d) S2p region of His-Au NCs and MES-Au NCs.

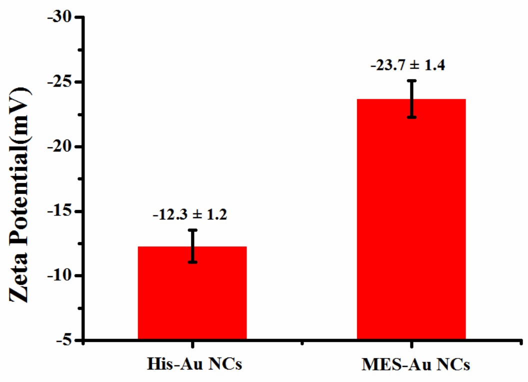

Figure S4. Zeta potential of His-Au NCs and MES-Au NCs.
